# Supplementary material for: Cellulose Acetate-Supported Copper as an Efficient Sustainable Heterogenous Catalyst for Azide-Alkyne Cycloaddition Click Reactions in Water
Source: Int J Mol Sci. 2023 May 26;24(11):9301. doi: 10.3390/ijms24119301 (PMC10253314; doi:10.3390/ijms24119301)
Supplement: Supplementary file 1 [file ijms-24-09301-s001.zip › ijms-2371794-supplementary.pdf]

Cellulose Acetate-Supported Copper as an Efficient Sustainable Heterogenous Catalyst for Azide-Alkyne Cycloaddition Click Reactions in Water

Salah-Eddine Stiriba [1,2,*], Lahoucine Bahsis [2], Elhouceine Benhadria [3], Khaoula Oudghiri [4], Moha Taourirte [4] and Miguel Julve [1]

[1] Instituto de Ciencia Molecular/ICMol, Universidad de Valencia, C/ Catedrático José Beltrán 2, 46980 Paterna, Valencia, Spain
[2] Laboratoire de Chimie Analytique et Moléculaire (LCAM), Faculté Polydisciplinaire de Safi, Université Cadi Ayyad, Safi 46030, Morocco
[3] Département de Chimie, Faculté des Sciences d'El Jadida, Université Chouaïb Doukkali, El Jadida 24000, Morocco
[4] Laboratoire de Recherche en Développement Durable et Santé, Faculté des Sciences et Techniques de Marrakech, Université Cadi Ayyad, Marrakech 40000, Morocco
* Correspondence: stiriba@uv.es

1-Benzyl-4-phenyl-1H-1,2,3-triazole (3a)

White solid. ^{1}H NMR (300MHz, $CDCl_3$, δ ppm): 5.59 (s, 2H, CH_2), 7.28-7.44 (m, 8H, CH_{ar}), 7.69 (s, 1H, $CH_{triazole}$), 7.81-7.84(d, 2H, CH_{ar}). ^{13}C NMR (75MHz, $CDCl_3$, δ ppm): 54.6 (CH_2); 120.0 (CH_{ar}); 126.1 (CH_{ar}); 128.5 (2 CH_{ar}); 128.6 (2 CH_{ar}); 129.2 (C_{ar}); 129.6 ($CH_{triazole}$); 130.9 (C_{ar}); 135.1 (C_{ar}).

1- Benzyl-4-p-tolyl-1H-1,2,3-triazole (3b)

White solid. ^{1}H NMR (300MHz, $CDCl_3$, δ ppm): 2.38 (s, 3H, CH_3), 5.59 (s, 2H, CH_2), 7.22-7.42 (m, 7H, CH_{ar}), 7.66 (s, 1H, $CH_{triazole}$), 7.70-7.73 (d, 2H, CH_{ar}). ^{13}C NMR (75MHz, $CDCl_3$, δ ppm): 21.7 (CH_3), 54.6 (CH_2), 126.0 (CH_{ar}), 128.2 (2CH_{ar}), 128.5 (2CH_{ar}), 129.2 (2CH_{ar}), 129.5 (2CH_{ar}), 129.9 (C_{ar}), 131.7 ($CH_{triazole}$), 135.1 (2C_{ar}), 138.4 ($C_{triazole}$).

1-benzyl-4-(phenoxymethyl)-1H-1,2,3-triazole (3c)

White solid. ^{1}H NMR (300MHz, $CDCl_3$, δ ppm): 5.21 (s, 2H, CH_2); 5.55 (s, 2H, OCH_2); 6.96-7.00 (m, 3H, CH_{ar}); 7.28-7.41 (m, 7H, CH_{ar}); 7.57 (s, 1H, $CH_{triazole}$). ^{13}C NMR (75MHz, $CDCl_3$, δ ppm): 54.7 (CH_2); 62.5 (CH_2); 115.2 (2 CH_{ar}); 116.6 (CH_{ar}); 121.7 ($CH_{triazole}$); 128.5 (CH_{ar}); 129.2 (2 CH_{ar}); 129.5 (4 CH_{ar}); 129.9 (C_{ar}); 134.9 ($C_{triazole}$); 158.6 (C_{ar}).

(1-benzyl-1H-1,2,3-triazol-4-yl)methyl benzoate (3d)

White solid. ^{1}H NMR (300MHz, $CDCl_3$, δ ppm): 5.47 (s, 2H, CH_2); 5.54 (s, 2H, CH_2); 7.28-7.38 (m, J=30, 5H, CH_{ar}); 7.40 (s, 1H, CH_{ar}); 7.43 (s, 1H, CH_{ar}); 7.46 (s, 1H, $CH_{triazole}$); 7.57 (s, 1H, CH_{ar}); 8.03-8.05 (d, 2H, CH_{ar}). ^{13}C NMR (75 MHz, $CDCl_3$, δ ppm) : 54.8 (CH_2); 58.4 (CH_2); 128.5 ($CH_{triazole}$); 128.8 (2 CH_{ar}); 129.3 (2 CH_{ar}); 129.6 (2 CH_{ar}); 130.1 (4 CH_{ar}); 133.6 (C_{ar}); 134.8 ($C_{triazole}$); 153.2 (C_{ar}); 166.8 ($C_{carbonyle}$).

1-benzyl-1H-1,2,3-triazole-4-carboxylic acid (3e)

White solid. ^{1}H NMR (300MHz, CD_3OD, δ ppm): 8.48 (s, 1H, $CH_{triazole}$); 7.33-7.40 (m, J=21 Hz, 5H, CH_{ar}); 5.94 (s, 1H, OH); 5.64 (s, 2H, CH_2). ^{13}C NMR (75MHz, CD_3OD, δ ppm): 55.6 (CH_2); 129.7-129.9 (5CH_{ar}); 130.2 ($CH_{triazole}$); 130.5 (C_{ar}); 136.7 ($C_{triazole}$); 162.3 ($C_{carbonyle}$).

4-((4-phenyl-1H-1,2,3-triazol-1-yl)methyl)pyridine (3f)

White solid. ^{1}H NMR (300MHz, $CDCl_3$, δ ppm): 5.73 (s, 2H, CH_2); 7.25-7.36 (m, 2H, CH_{ar}); 7.41-7.46 (m, 3H, CH_{ar}); 7.69-7.75 (m, 1H, CH_{ar}); 7.83-7.87 (m, 1H, CH_{ar}); 7.96 (s, 1H, $CH_{triazole}$); 8.64

(s, 2H, CH_{ar}). ^{13}C NMR (75 MHz, $CDCl_3$, δ ppm): 56.1 (CH_2); 120.6 (2 CH_{ar}); 124.0 (2 CH_{ar}); 126.1 (CH_{ar}); 128.6 (2 CH_{ar}); 129.2 (C_{ar}); 130.9 ($CH_{triazole}$); 137.8 (C_{ar}); 150.1 ($C_{triazole}$); 154.9 (2 CH_{ar}).

4-((4-(4-methoxyphenyl)-1H-1,2,3-triazol-1-yl)methyl)pyridine (3g)

White solid. ^{1}H NMR (300MHz, $CDCl_3$, δ ppm): 3.85 (s, 3H, CH_3); 5.51 (s, 2H, CH_2); 6.95-6.98 (d, 2H, CH_{ar}); 7.70-7.72 (d, 2H, CH_{ar}); 7.76-7.79 (d, 2H, CH_{ar}); 7.87 (s, 1H, $CH_{triazole}$); 8.63 (s, 2H, CH_{ar}). ^{13}C NMR (75 MHz, $CDCl_3$, δ ppm): 55.7 (CH_2); 56.1 (CH_3); 114.6 (2 CH_{ar}); 119.8 (C_{ar}); 123.7 (2 CH_{ar}); 127.4 (2 CH_{ar}); 130.7 ($CH_{triazole}$); 137.8 (C_{ar}); 148.5 ($C_{triazole}$); 154.9 (2 CH_{ar}); 160.0 (O-C_{ar}).

1-(4-methoxyphenyl)-4-phenyl -1H-1,2,3-triazole (3h)

White solid. ^{1}H NMR (300MHz, $CDCl_3$, δ ppm): 3.90 (s, 3H, OCH_3); 7.05-7.08 (d, 2H, CH_{ar}); 7.40-7.49 (m, 5H, CH_{ar}); 7.51-7.97 (d, 2H, CH_{ar}); 8.26 (s, 1H, $CH_{triazole}$). ^{13}C NMR (75MHz, $CDCl_3$, δ ppm): 56.1 (CH_3); 115.0 (CH_{ar}); 115.3 (C_{ar}); 122.7 (2 CH_{ar}); 126.3 (CH_{ar}); 126.6 (2CH_{ar}); 127.0 (2CH_{ar}); 129.0 ($CH_{triazole}$); 129.4 (C_{ar}); 146.1 ($C_{triazole}$); 160.5 (C_{ar}).

1-(4-isopropylphenyl)-4-phenyl-1H-1,2,3-triazole (3i)

White solid. ^{1}H NMR (400MHz, DMSO, δ ppm) : 1.24 (s, 3H,CH_3); 1.26 (s, 3H,CH_3); 2.95-3.04 (m, 1H, CH); 7.36-7.40 (t, 1H, CH_{ar}); 7.48-7.50 (d, 2H, CH_{ar}); 7.51-7.52 (d, 2H, CH_{ar}); 7.84-7.87 (d, 2H, CH_{ar}); 7.94-7.96 (d, 2H, CH_{ar}); 9.24 (s, 1H, $CH_{triazole}$). ^{13}C NMR (100MHz, DMSO, δ ppm): 24.1 (2CH_3); 33.5 (CH); 119.9 (C_{ar}); 120.4 (2CH_{ar}); 125.7 (2CH_{ar}); 128.1 (CH_{ar}); 128.5 (2CH_{ar}); 129.3 (2CH_{ar}); 130.7 ($CH_{triazole}$); 130.5 (C_{ar}); 148.0 ($C_{triazole}$); 149.5 (C_{ar}).

1-Benzyl-4-phenyl-1H-1,2,3-triazole (3a)

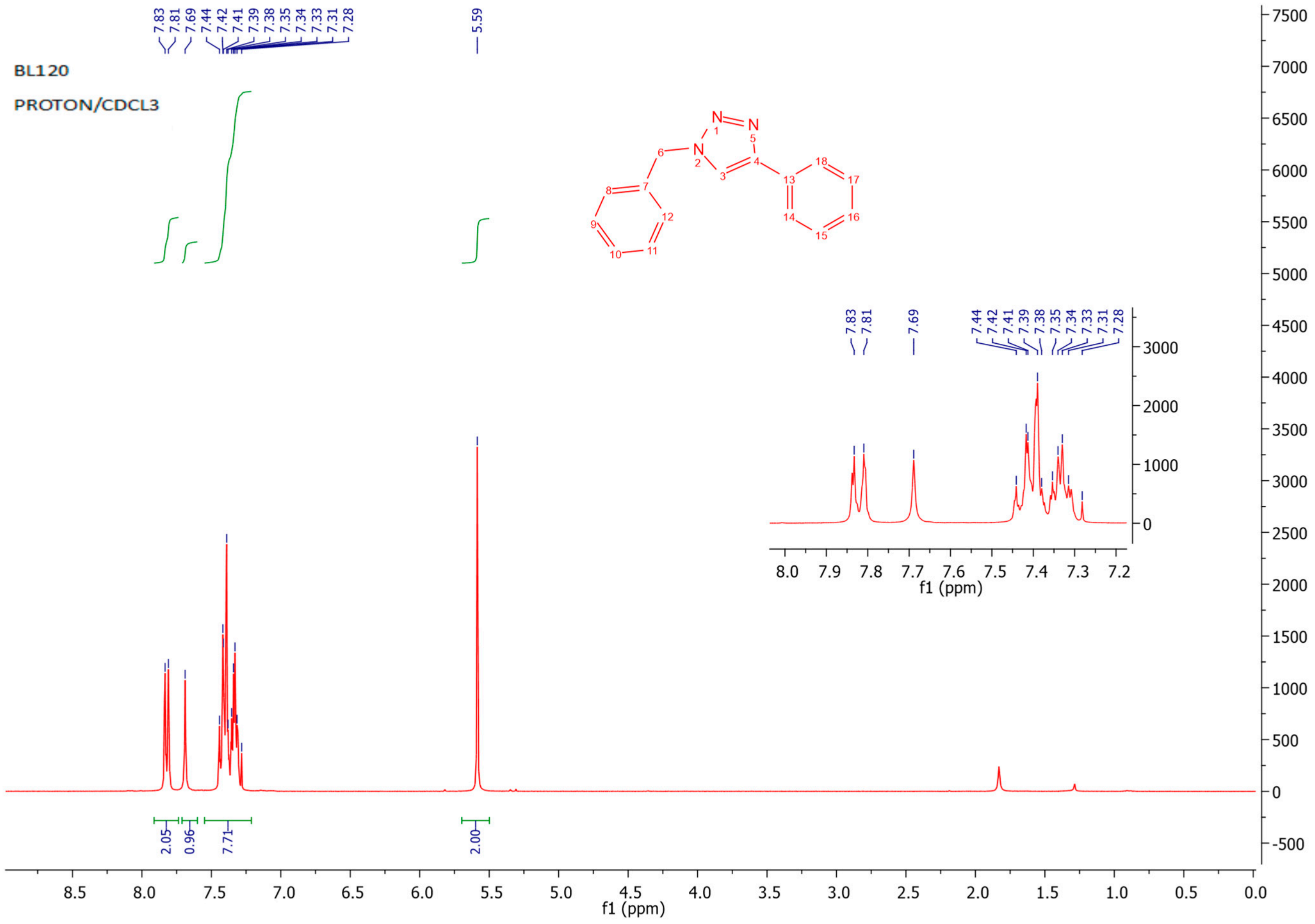

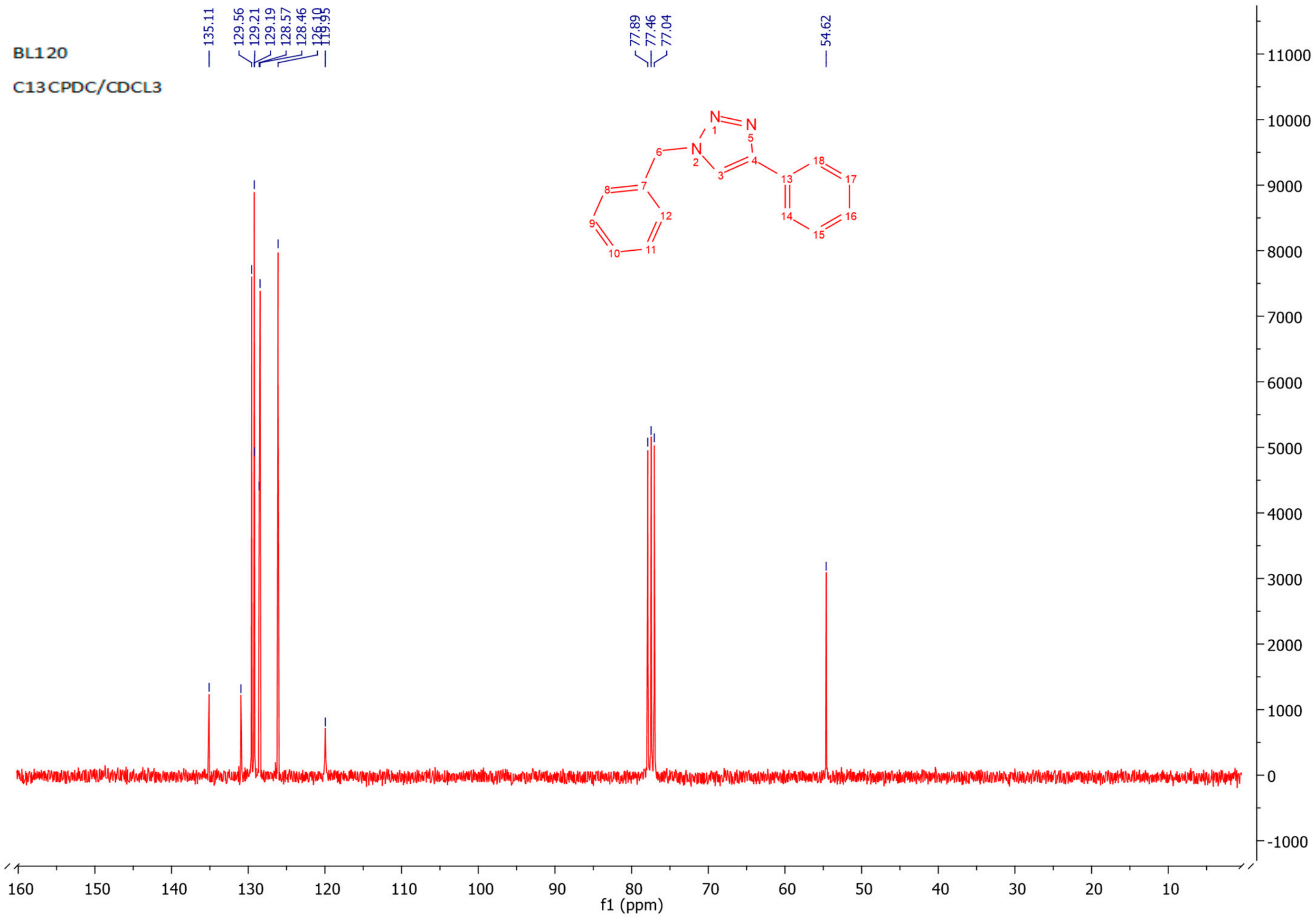
BL120
C13CPDC/CDCL3
135.11
129.56
129.21
129.19
128.57
128.46
126.10
119.95
77.89
77.46
77.04
54.62
f1 (ppm)

1-(4-methylbenzyl)-4-phenyl-1H-1,2,3-triazole (3b)

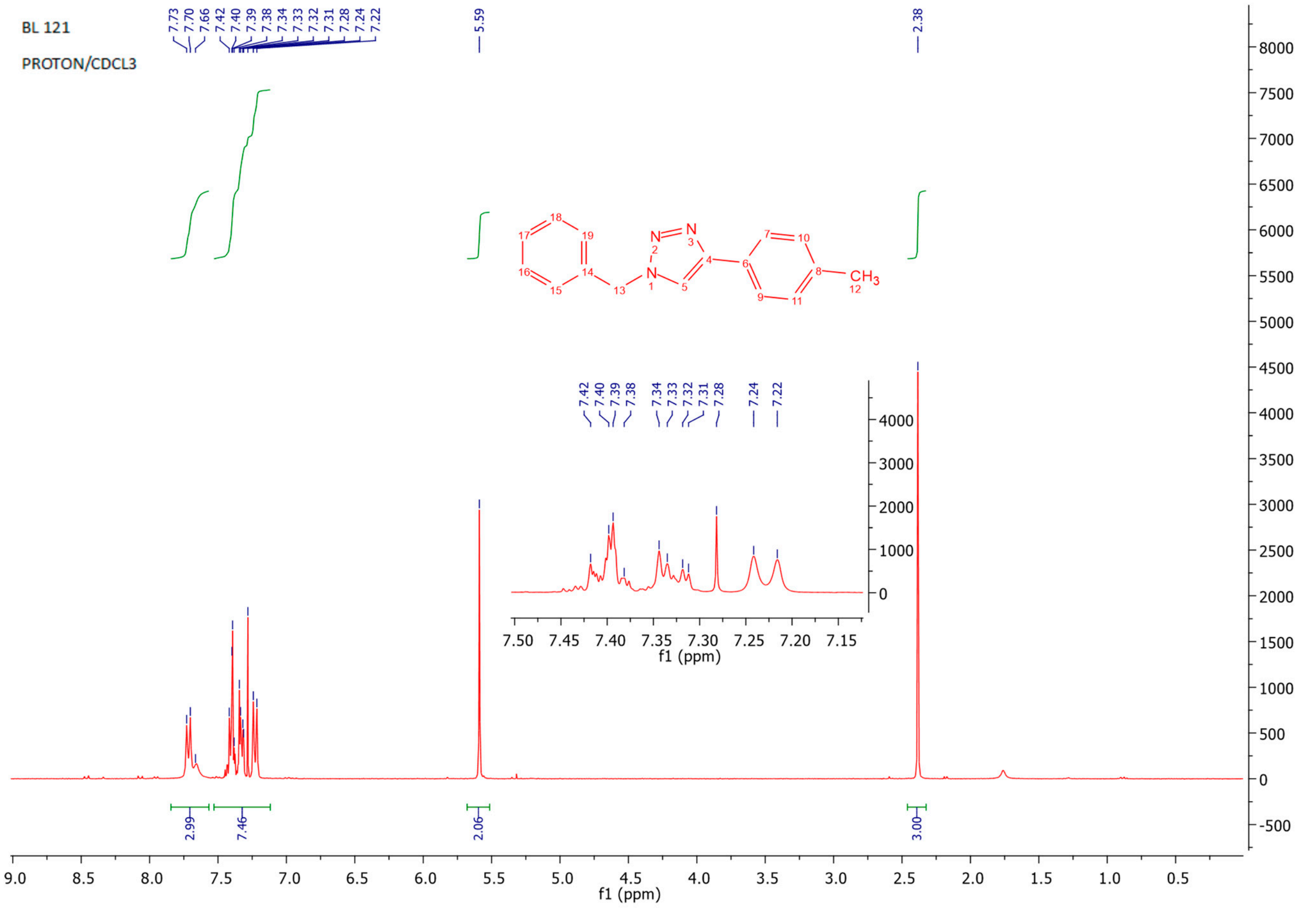

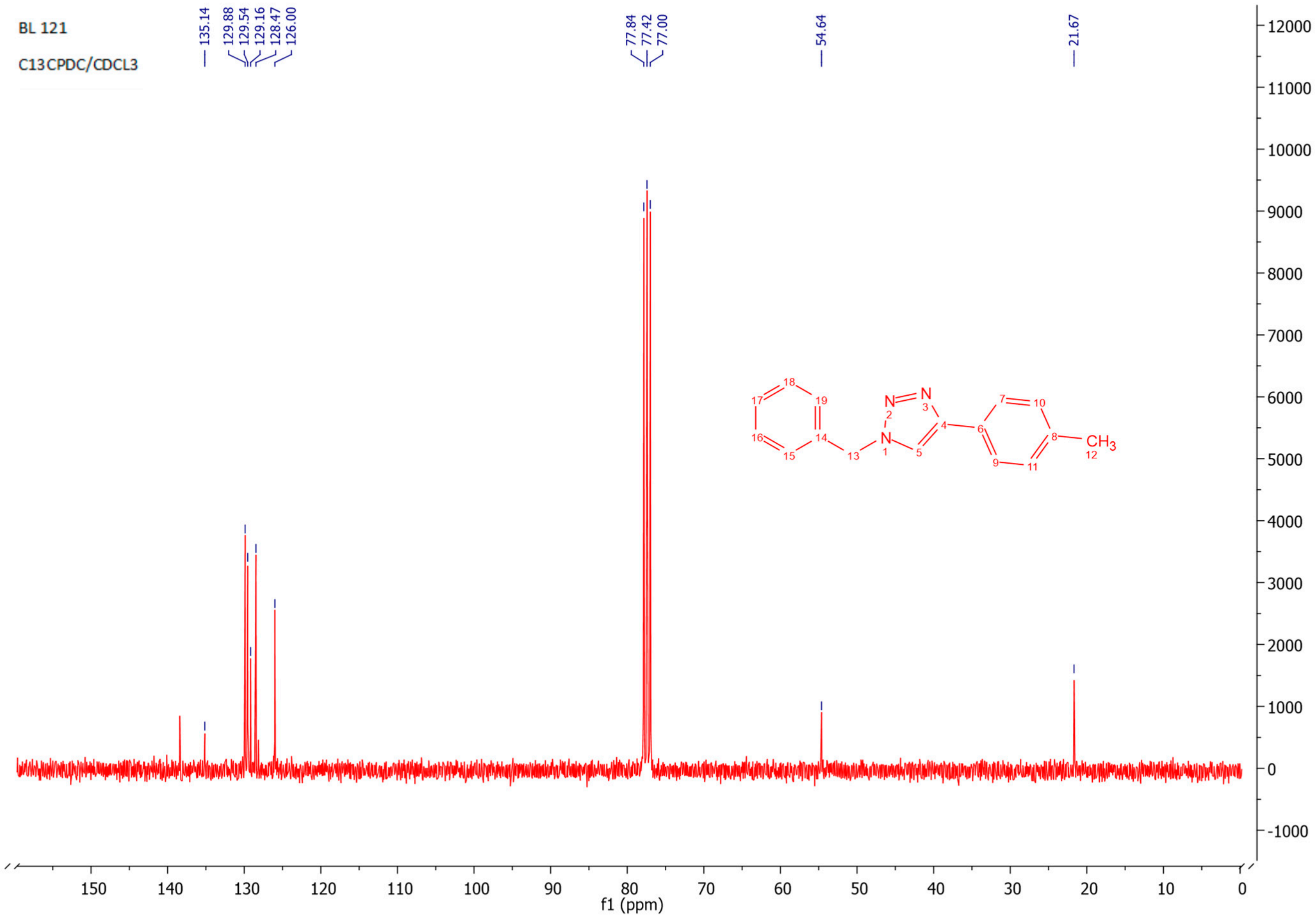
BL 121
C13CPDC/CDCL3
135.14
129.88
129.54
129.16
128.47
126.00
77.84
77.42
77.00
54.64
21.67
f1 (ppm)
CH3

1-benzyl-4-(phenoxymethyl)-1H-1,2,3-triazole (3c)

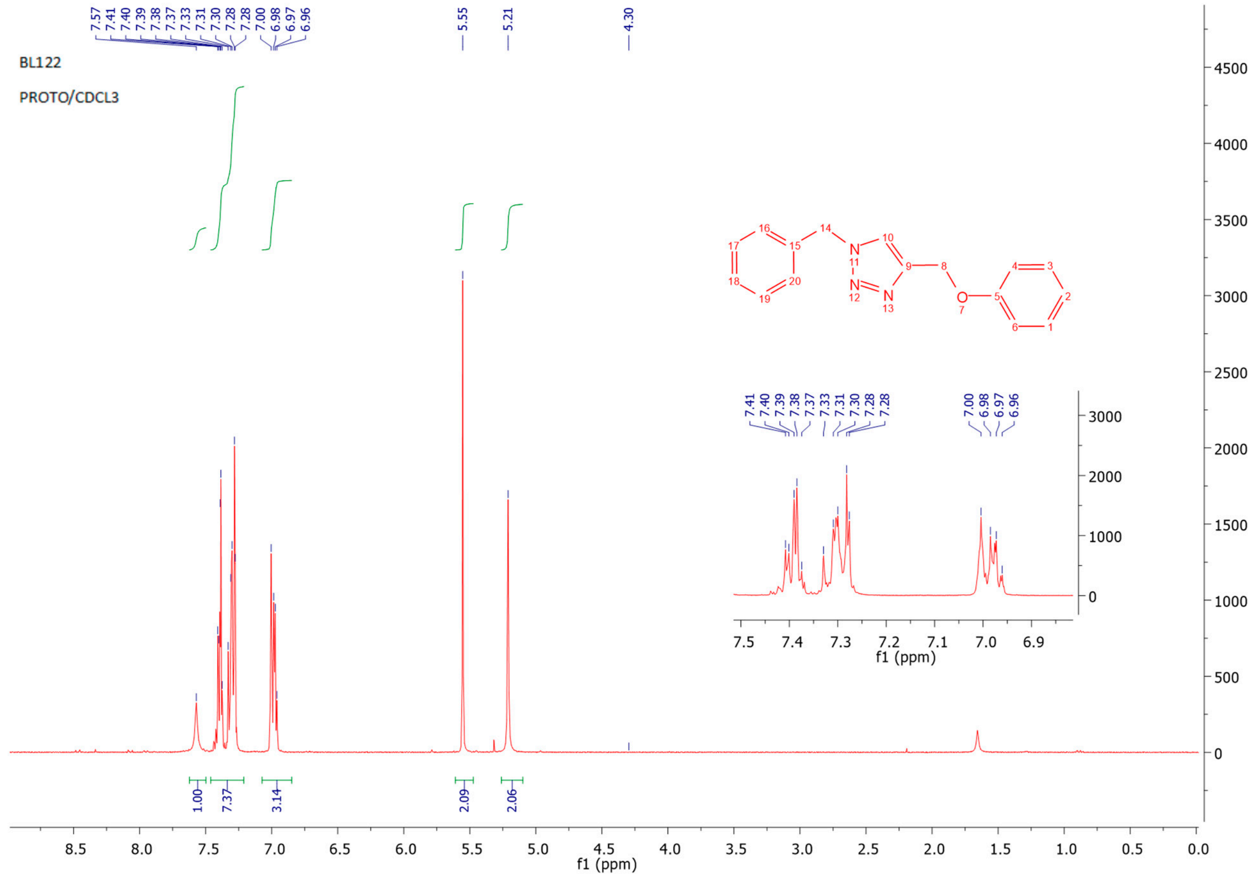

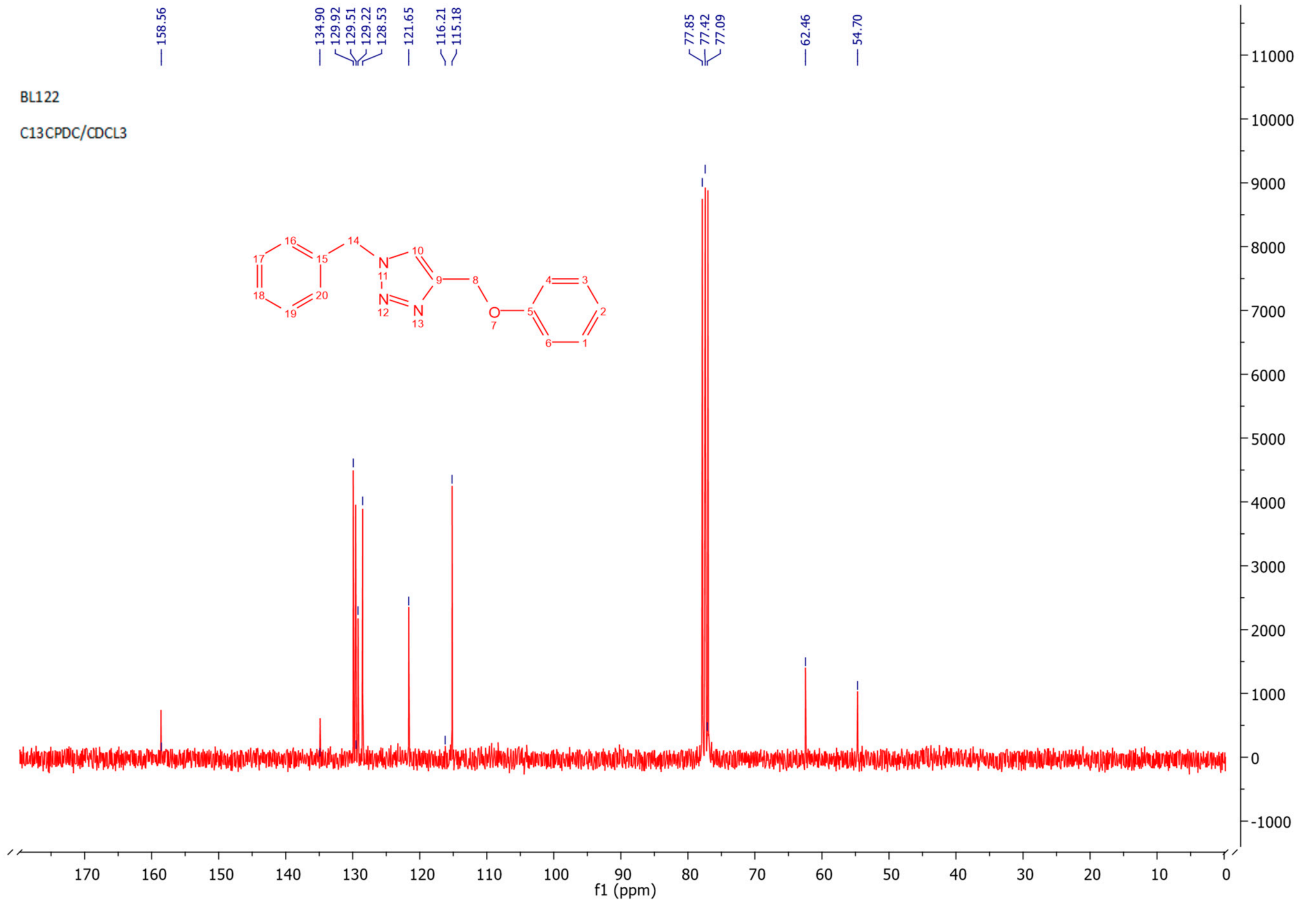

158.56
134.90
129.92
129.51
129.22
128.53
121.65
116.21
115.18
77.85
77.42
77.09
62.46
54.70
BL122
C13CPDC/CDCL3
f1 (ppm)

(1-benzyl-1H-1,2,3-triazol-4-yl)methyl benzoate (3d)

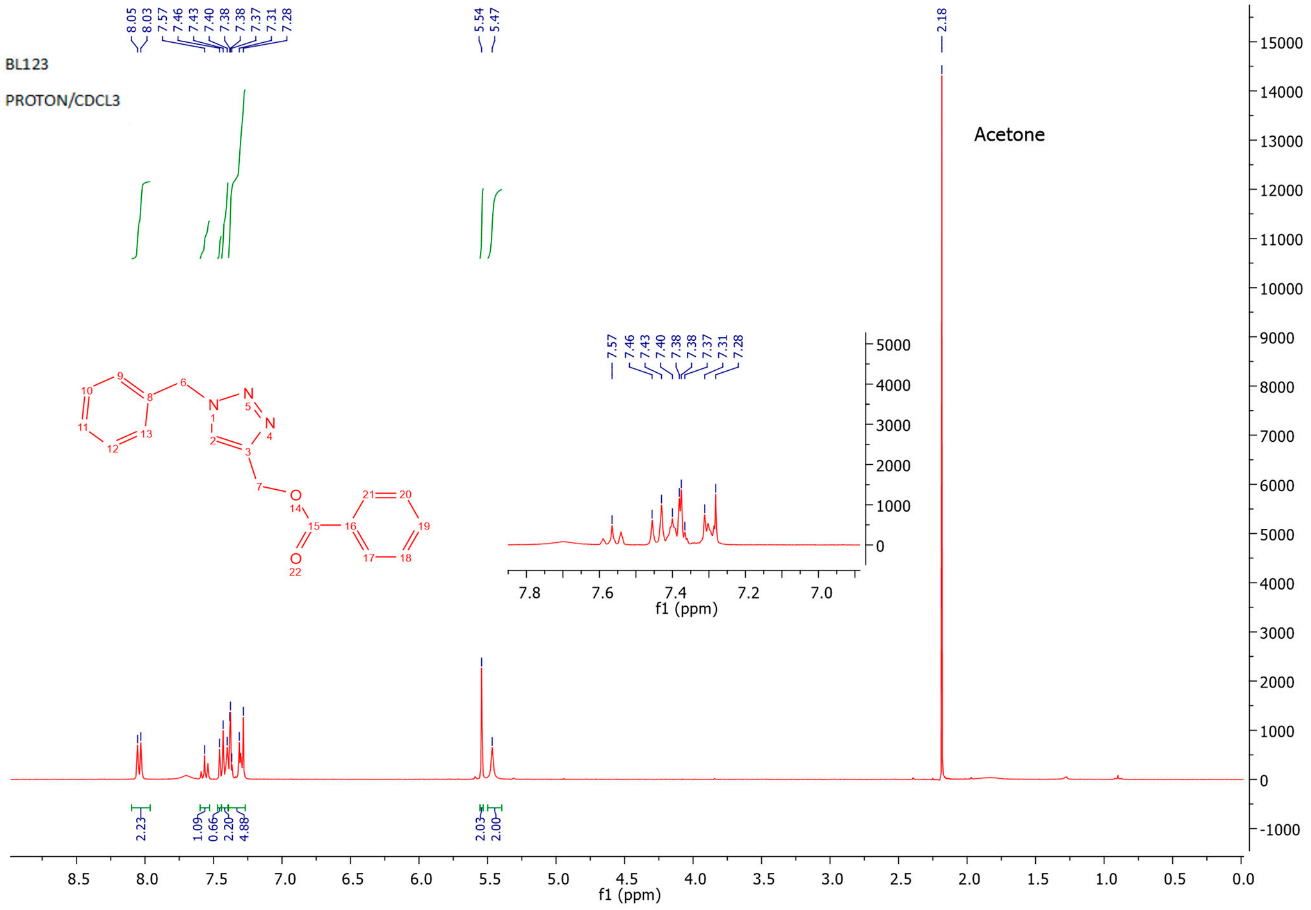

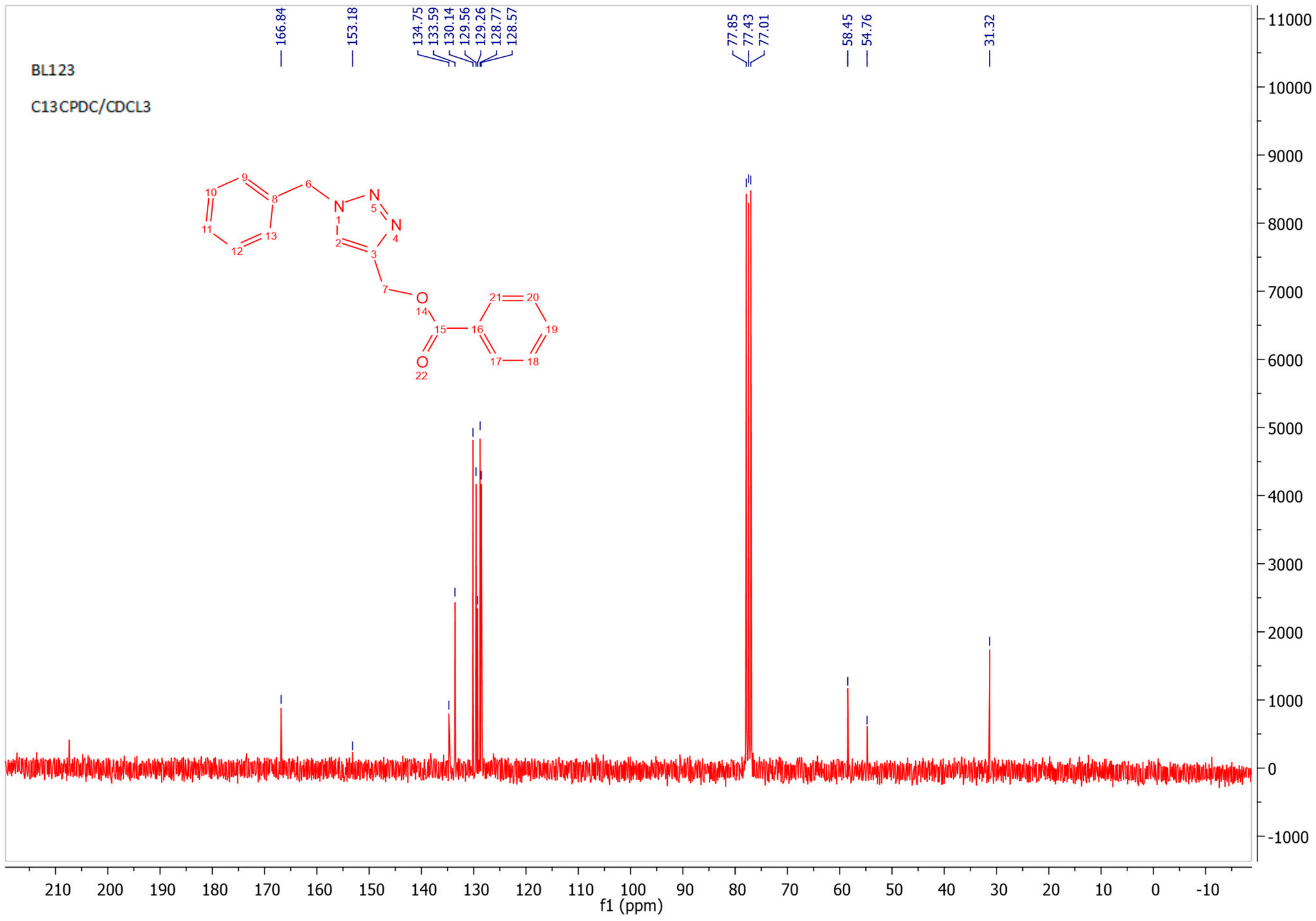

BL123
C13CPDC/CDCL3
166.84
153.18
134.75
133.59
130.14
129.56
129.26
128.77
128.57
77.85
77.43
77.01
58.45
54.76
31.32
f1 (ppm)

1-benzyl-1H-1,2,3-triazole-4-carboxylic acid (3e)

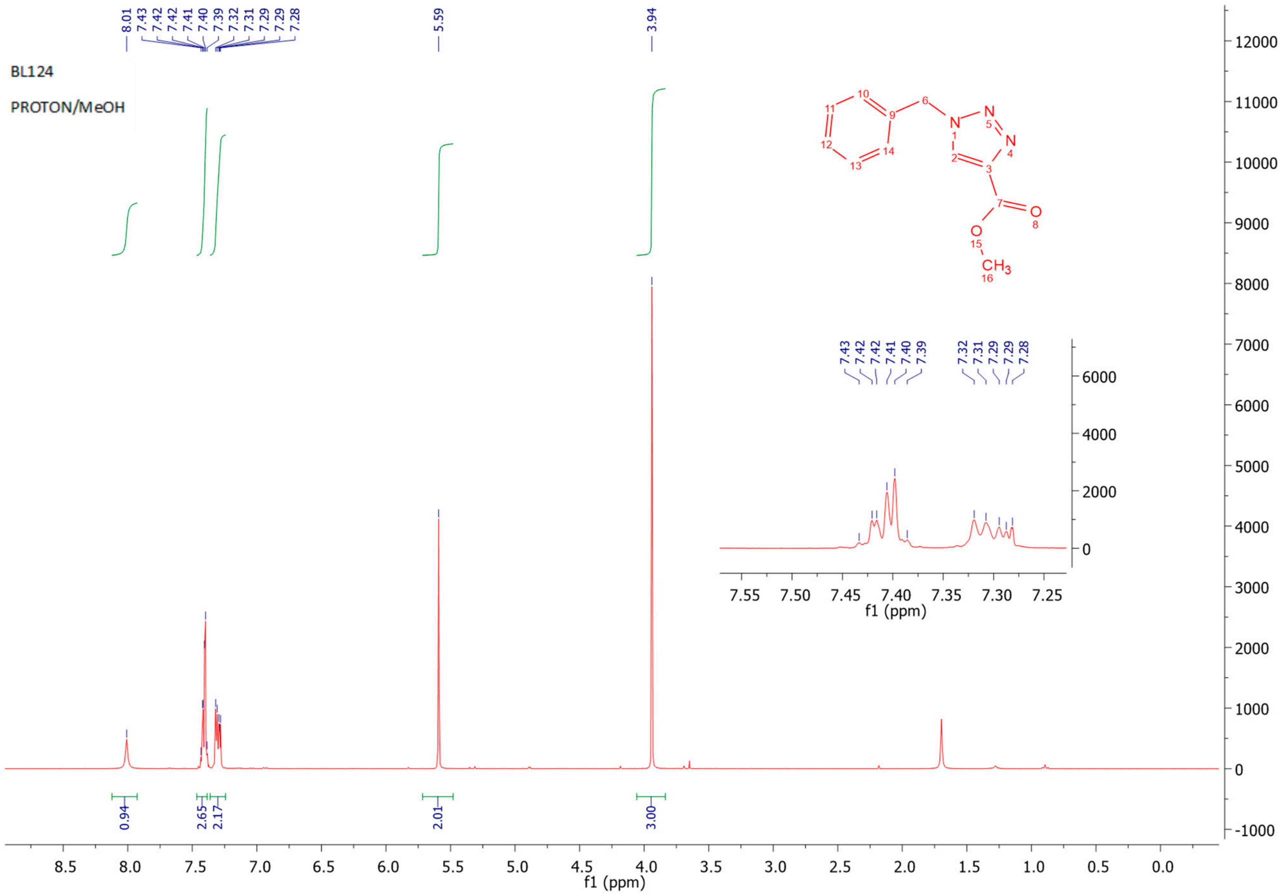

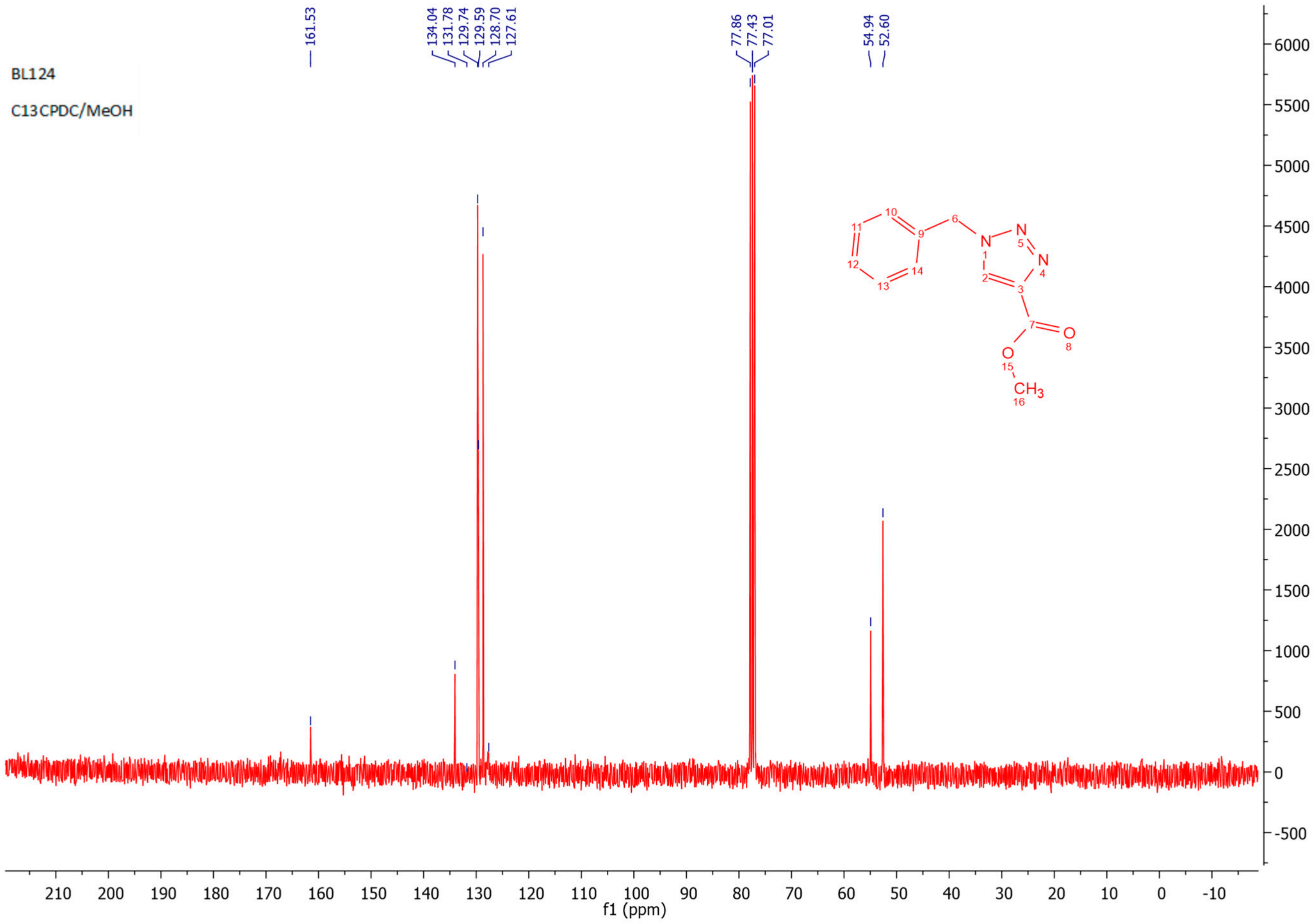
161.53
134.04
131.78
129.74
129.59
128.70
127.61
77.86
77.43
77.01
54.94
52.60
BL124
C13CPDC/MeOH
f1 (ppm)

4-((4-phenyl-1H-1,2,3-triazol-1-yl)methyl)pyridine (3f)

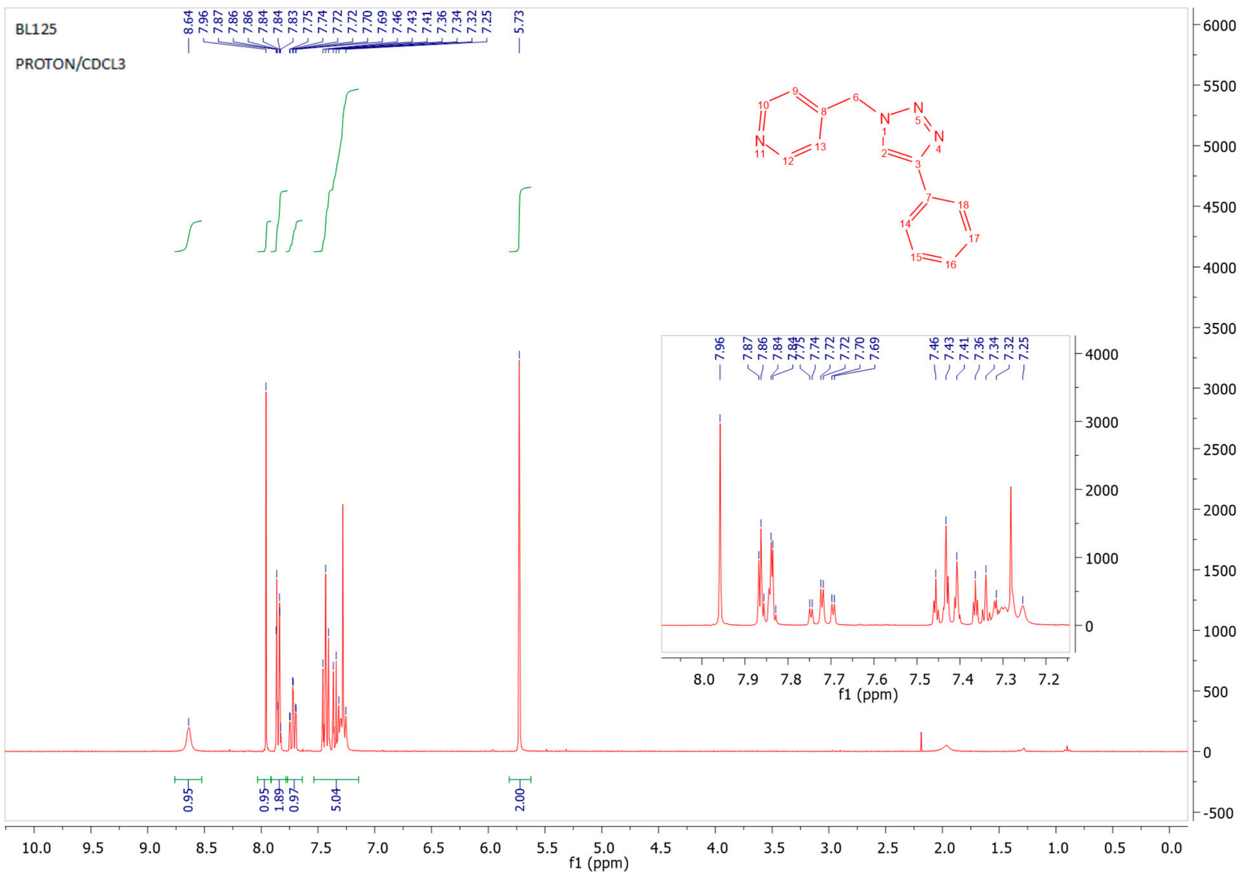

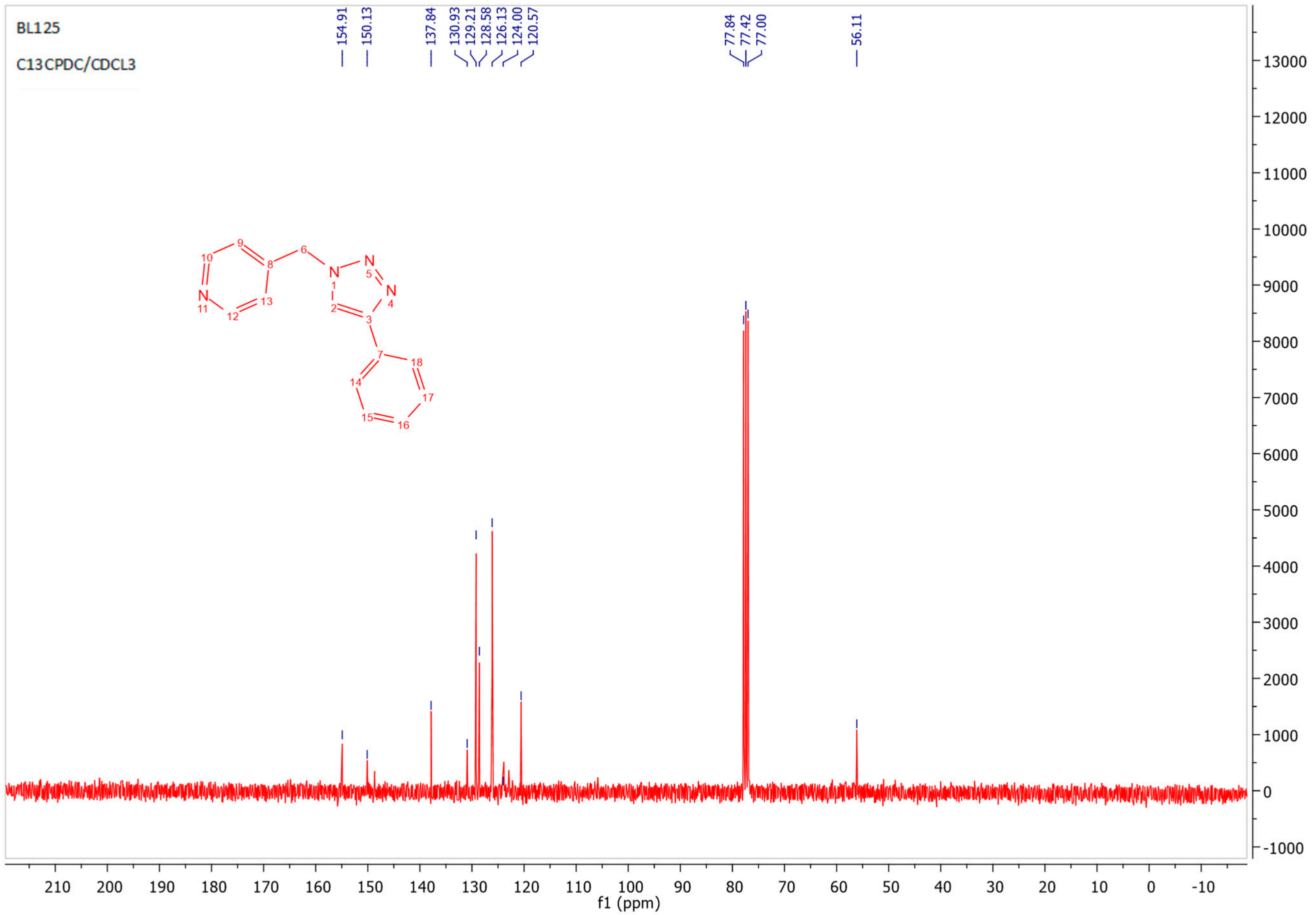
BL125
C13CPDC/CDCL3
154.91
150.13
137.84
130.93
129.21
128.58
126.13
124.00
120.57
77.84
77.42
77.00
56.11
f1 (ppm)

4-((4-(4-methoxyphenyl)-1H-1,2,3-triazol-1-yl)methyl)pyridine (3g)

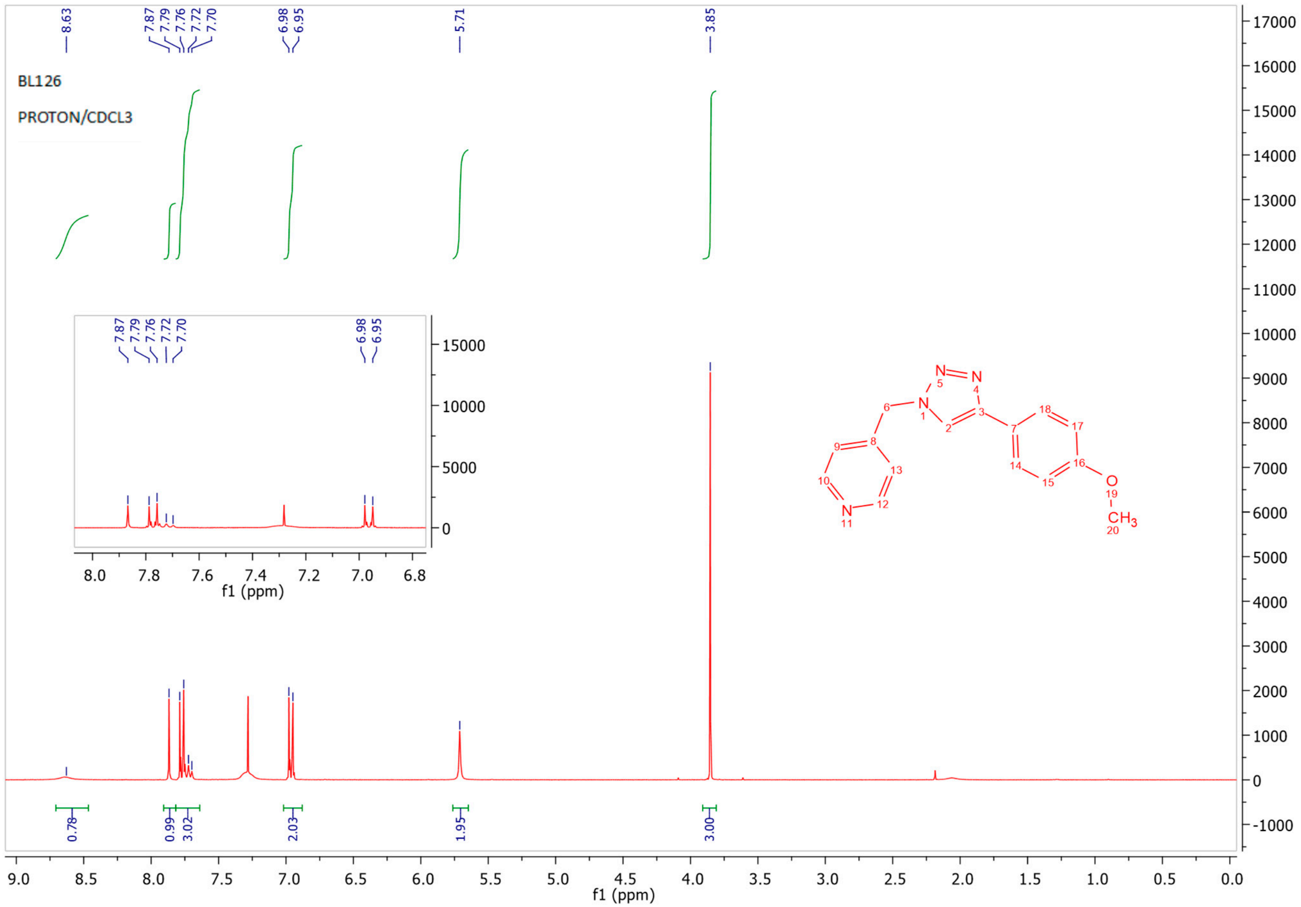

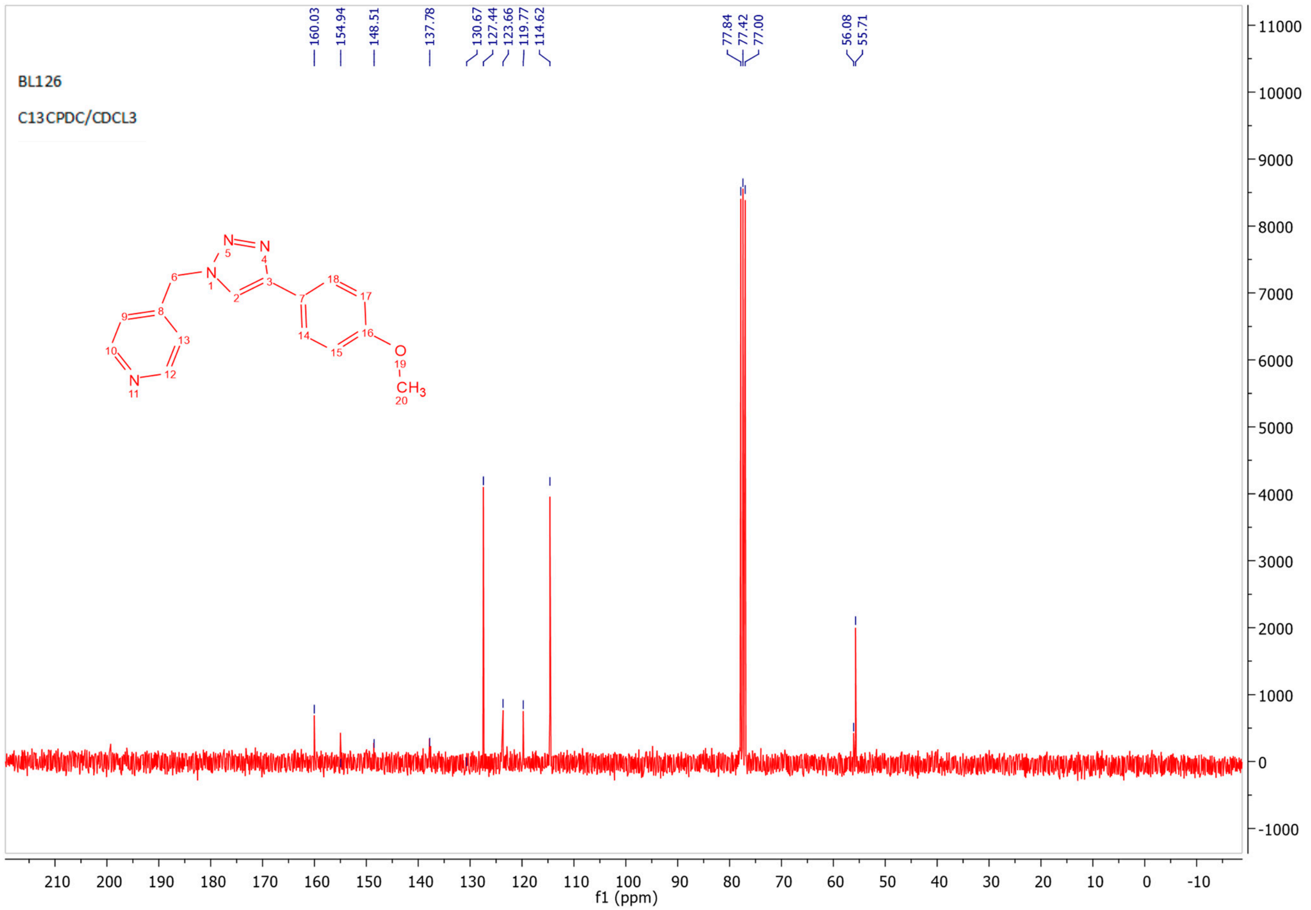

BL126
C13CPDC/CDCL3
160.03
154.94
148.51
137.78
130.67
127.44
123.66
119.77
114.62
77.84
77.42
77.00
56.08
55.71
f1 (ppm)

1-(4-methoxyphenyl)-4-phenyl-1H-1,2,3-triazole (3h)

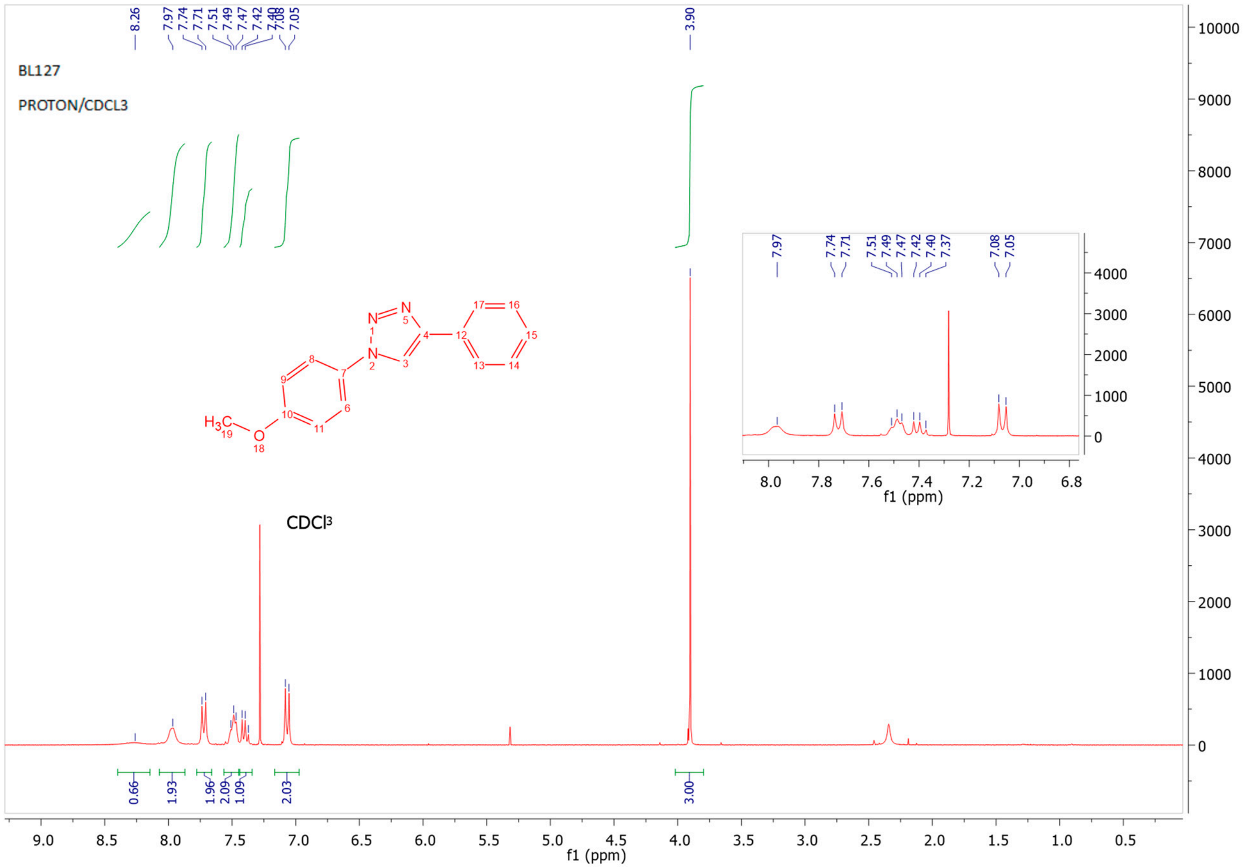

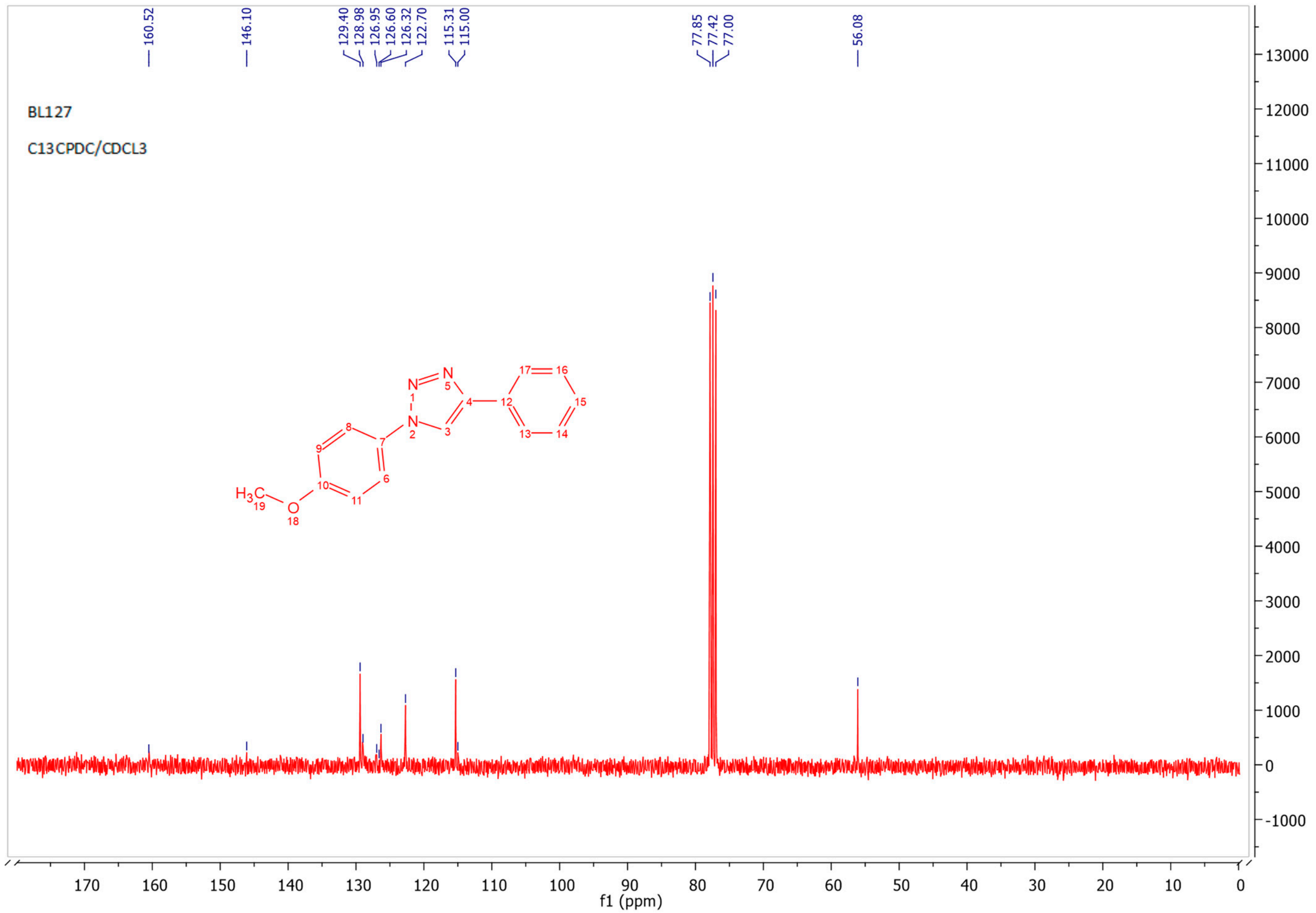

160.52
146.10
129.40
128.98
126.95
126.60
126.32
122.70
115.31
115.00
77.85
77.42
77.00
56.08
BL127
C13CPDC/CDCL3
f1 (ppm)

1-(4-isopropylphenyl)-4-phenyl-1H-1,2,3-triazole (3i)

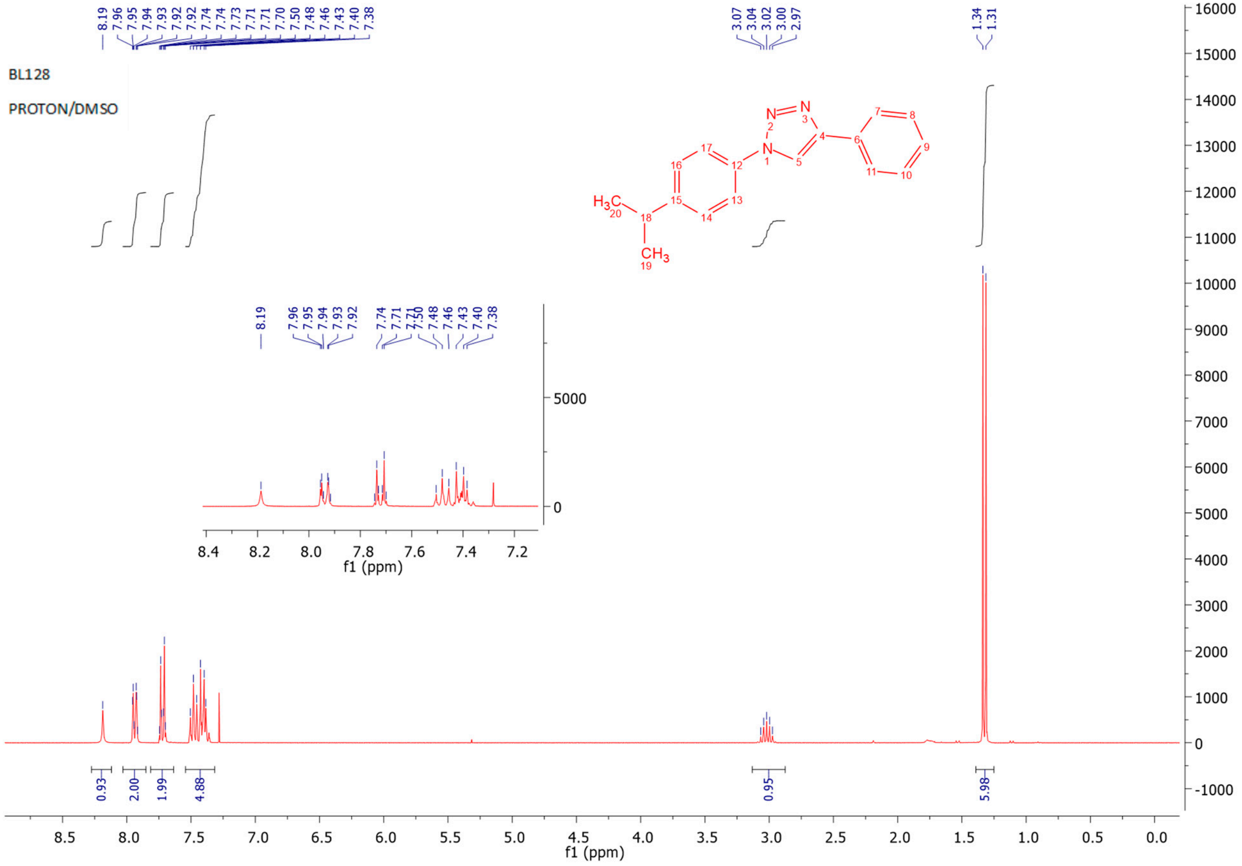

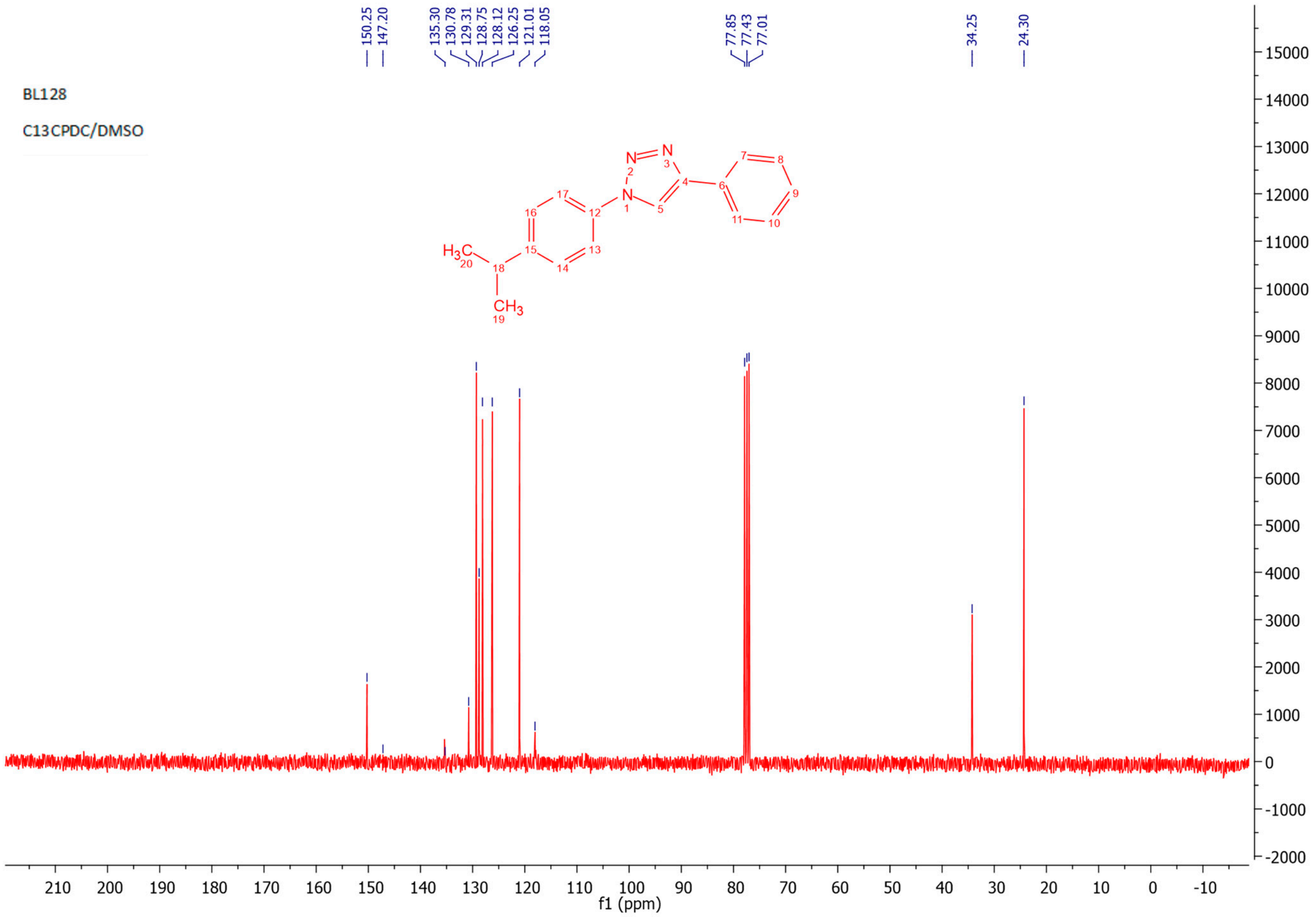

BL128
C13CPDC/DMSO
150.25
147.20
135.30
130.78
129.31
128.75
128.12
126.25
121.01
118.05
77.85
77.43
77.01
34.25
24.30
f1 (ppm)
